# Deletion at *ITPR1* Underlies Ataxia in Mice and Spinocerebellar Ataxia 15 in Humans

**DOI:** 10.1371/journal.pgen.0030108

**Published:** 2007-06-22

**Authors:** Joyce van de Leemput, Jayanth Chandran, Melanie A Knight, Lynne A Holtzclaw, Sonja Scholz, Mark R Cookson, Henry Houlden, Katrina Gwinn-Hardy, Hon-Chung Fung, Xian Lin, Dena Hernandez, Javier Simon-Sanchez, Nick W Wood, Paola Giunti, Ian Rafferty, John Hardy, Elsdon Storey, R. J. McKinlay Gardner, Susan M Forrest, Elizabeth M. C Fisher, James T Russell, Huaibin Cai, Andrew B Singleton

**Affiliations:** 1 Molecular Genetics Unit, Laboratory of Neurogenetics, National Institute on Aging, National Institutes of Health, Bethesda, Maryland, United States of America; 2 Department of Neurodegenerative Disease, Institute of Neurology, Queen Square, London, United Kingdom; 3 Transgenics Unit, Laboratory of Neurogenetics, National Institute on Aging, National Institutes of Health, Bethesda, Maryland, United States of America; 4 Department of Biology, Johns Hopkins University, Baltimore, Maryland, United States of America; 5 Neurogenetics Branch, National Institute of Neurological Disorders and Stroke, National Institutes of Health, Bethesda, Maryland, United States of America; 6 Section on Cell Biology and Signal Transduction, National Institute on Child Health and Development, National Institutes of Health, Bethesda, Maryland, United States of America; 7 Reta Lila Weston Institute of Neurological Studies, University College London, London, United Kingdom; 8 Cell Biology and Gene Expression Unit, Laboratory of Neurogenetics, National Institute on Aging, National Institutes of Health, Bethesda, Maryland, United States of America; 9 Department of Molecular Neuroscience, Institute of Neurology, Queen Square, London, United Kingdom; 10 National Institute of Neurological Disorders and Stroke, National Institutes of Health, Bethesda, Maryland, United States of America; 11 Laboratory of Neurogenetics, National Institute on Aging, National Institutes of Health, Bethesda, Maryland, United States of America; 12 Department of Neurology, Chang Gung Memorial Hospital and College of Medicine, Chang Gung University, Taipei, Taiwan; 13 Unitat de Genética Molecular, Departamento de Genómica y Proteómica, Instituto de Biomedicina de Valencia, Consejo Superior de Investigaciones Científicas, Valencia, Spain; 14 Department of Medicine, Alfred Hospital, Monash University, Melbourne, Australia; 15 Genetic Health Services Victoria, Melbourne, Australia; 16 Murdoch Childrens Research Institute, Royal Children's Hospital, Melbourne, Australia; 17 Australian Genome Research Facility, Walter and Eliza Hall Institute of Medical Research, Melbourne, Australia; University of Minnesota, United States of America

## Abstract

We observed a severe autosomal recessive movement disorder in mice used within our laboratory. We pursued a series of experiments to define the genetic lesion underlying this disorder and to identify a cognate disease in humans with mutation at the same locus. Through linkage and sequence analysis we show here that this disorder is caused by a homozygous in-frame 18-bp deletion in *Itpr1* (*Itpr1^Δ18/Δ18^*), encoding inositol 1,4,5-triphosphate receptor 1. A previously reported spontaneous *Itpr1* mutation in mice causes a phenotype identical to that observed here. In both models in-frame deletion within *Itpr1* leads to a decrease in the normally high level of *Itpr1* expression in cerebellar Purkinje cells. Spinocerebellar ataxia 15 (SCA15), a human autosomal dominant disorder, maps to the genomic region containing *ITPR1;* however, to date no causal mutations had been identified. Because ataxia is a prominent feature in *Itpr1* mutant mice, we performed a series of experiments to test the hypothesis that mutation at *ITPR1* may be the cause of SCA15. We show here that heterozygous deletion of the 5′ part of the *ITPR1* gene, encompassing exons 1–10, 1–40, and 1–44 in three studied families, underlies SCA15 in humans.

## Author Summary

We have identified a spontaneous in-frame deletion mutation in the gene *Itpr1* that causes a recessive movement disorder in mice. In an attempt to define whether any similar disease occurs in humans we performed a literature search for diseases linked to the human chromosomal region containing *ITPR1*. We identified the disease spinocerebellar ataxia 15 as linked to this region. High-density genomic analysis of affected members from three families revealed that disease in these patients was caused by deletion of a large portion of the region containing *ITPR1*. We show here that this mutation results in a dramatic reduction in ITPR1 in cells from these patients. These data show convincingly that *ITPR1* deletion underlies spinocerebellar ataxia 15 in humans.

## Introduction

The use of forward genetics to define novel loci of interest in human disease has become increasingly viable with the implementation of large-scale mutagenesis programs. Prior to these initiatives this work was carried out in part by the investigation of spontaneous mutations that cause disorders in mouse breeding colonies. Careful observation of these serendipitous events has led to the establishment and study of many in vivo disease models [3].

During the generation of a knockout line of mice we noted an early movement disorder that was inherited independently of targeting vector transmission. We embarked on a series of experiments to identify the genetic lesion underlying this movement disorder and to identify a cognate disease and corresponding mutation in humans. Here we describe this effort and the discovery of deletion at the *ITPR1* locus as a cause of this disorder in mice and of spinocerebellar ataxia 15 (SCA15) in humans.

## Results/Discussion

During the generation of a line of mice with knockout of the gene *Park7* we noted an early movement disorder that was inherited independently of targeting vector transmission. Our initial observations suggested the affected mice suffered from an apparently paroxysmal movement disorder, often induced by touch. The abnormal movements occurred predominantly below the cervical level, and the disorder appeared progressive. At initial examination, a human movement disorder specialist (K. G.-H.) likened the disorder to episodic intermittent ataxia or kinesiogenic paroxysmal dystonia and predicted the involvement of an ion channel mutation in the etiology. Affected mice presented at approximately postnatal day 14, and survival time without weaning was on average 4 wk after onset.

Breeding experiments suggested that the observed disorder was inherited in an autosomal recessive manner. To map the location of the disease-causing lesion, we performed genome-wide linkage analysis using strain-specific single nucleotide polymorphisms (SNPs) at 120 loci across the mouse genome. Analysis of these data showed a single genomic region with significant linkage to disease, providing a two-point LOD score of 5.13 at marker 20.MMHAP85FLG2 on Chromosome 6qE1. The linked haplotype suggested the mutation had occurred on the 129x1/SvJ background (Figure S1).

**Figure 1:**
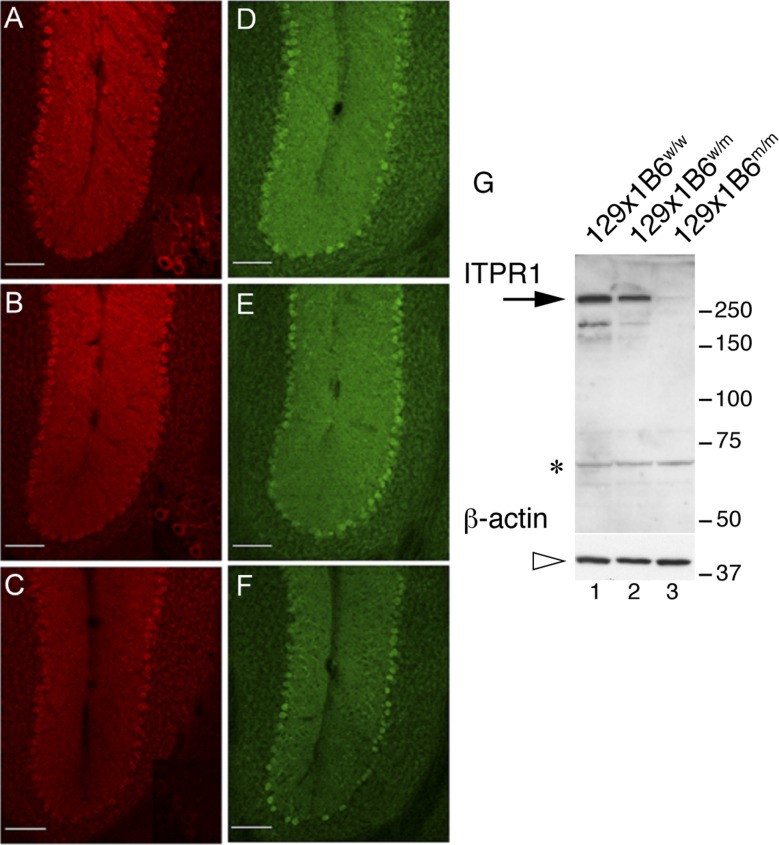
Immunohistochemistry and Western Blot Analysis of ITPR1 Protein Levels in Mouse Cerebellum (A–F) Immunohistochemistry of cerebellum from a wild-type mouse (A and D), a mouse heterozygous for the *Itpr1* 18-bp deletion (B and E), and a mouse homozygous for the 18-bp *Itpr1* deletion (C and F). (A–C) Immunohistochemistry using polyclonal Itpr1 anti-rabbit antibody (1:2,000; Alexa Fluor 555); (D–F) immunohistochemistry using monoclonal Calb1 anti-mouse antibody (1:6,000; Alexa Fluor 488). Scale bars denote 100 μm. As previously described, Iptr1 is highly expressed in the Purkinje cells. Notably, there appears to be decreased immunoreactivity to Itpr1 in the heterozygous and homozygous mutant mice. (G) Western blot performed to examine Itpr1 levels in whole brain from wild-type, *Itpr1^wt/Δ18^,* and *Itpr1^Δ18/Δ18^* mice; this clearly shows a reduction of Itpr1 in brain tissue from *Itpr1^wt/Δ18^* mice and a greater reduction of Itpr1 in *Itpr1^Δ18/Δ18^* mice.

Literature searches revealed that among disease lines mapped to 6qE1, the spontaneous mutant *opt* mouse displays a strikingly similar presentation to that described here [1]. The underlying genetic lesion causing the *opt* phenotype is a homozygous in-frame deletion of exons 43 and 44 of the gene *Itpr1* (*Itpr1^opt^* ^/*opt*^), encoding inositol 1,4,5-triphosphate receptor 1 (Itpr1). Sequencing of all exons and intron–exon boundaries of *Itpr1* in affected mice from the current study revealed a single mutation within *Itpr1:* a novel in-frame deletion of 18 bp within exon 36 (*Itpr1^Δ18/Δ18^*). To confirm the pathogenicity of this mutation we crossed heterozygous mice from the current study (*Itpr1^wt/Δ18^*) with mice heterozygous for the *opt* mutation (*Itpr1^wt/opt^*). This resulted in two litters of mice with a total of four affected *Itpr1^opt/Δ18^* pups (from a total of 15) with a phenotype indistinguishable from that of the *Itpr1^Δ18/Δ18^* and *Itpr1^opt^* ^/*opt*^ mice [1]. Furthermore, this phenotype was similar, although less severe, to that described in a mouse line with targeted deletion of *Itpr1,* where ataxia was described as a prominent feature [4]. As with the *Itpr1^opt^* ^/*opt*^ mice, where the deletion of exons 43 and 44 is also predicted to leave the translational reading frame unaffected, the in-frame *Itpr1^Δ18/Δ18^* deletion mutation results in markedly decreased levels of Itpr1 in cerebellar Purkinje cells. In these two spontaneous mutants [1] and in the *Itpr1-*deficient mouse [4] generated by gene targeting, decreased Itpr1 expression is associated with the same autosomal recessive movement disorder (Figure 1).

Given our interest in human neurological disease we sought to identify any cognate human disorders where linkage had been established to the syntenic region of the human genome, but where no causal mutation had been identified. SCA15, an adult-onset autosomal dominant progressive ataxia is linked to this locus [5]. Although missense mutation of *ITPR1* had previously been ruled out [2] and the mode of inheritance was inconsistent with that seen in the *Itpr1^Δ18^* and *Itpr1^opt^* mice, the phenotypic presence of ataxia in the mice led us to reexamine this candidate gene as a possible cause of SCA15.

**Figure 2:**
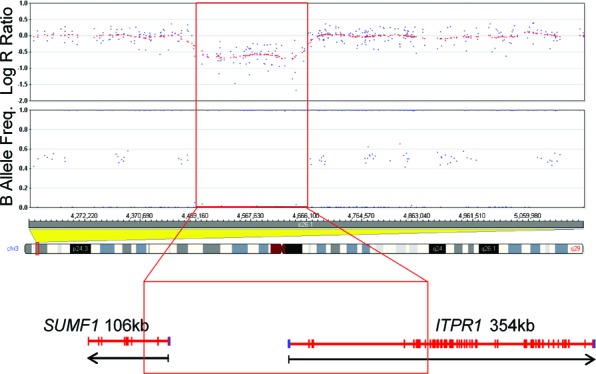
Metrics Derived from Analysis of DNA from Affected Family Member 7 Using Illumina Infinium HumanHap550 Genotyping Chips The upper and lower plots are log R ratio and B allele frequency, respectively, at an ~800-kb segment on the p arm of Chromosome 3. Log R ratio is the ratio of normalized, observed R to expected R for each SNP (each SNP is a blue dot) and thus serves as a surrogate of copy number at each locus. B allele frequency is a measure of the number of times the A or B alleles are detected at each locus (each SNP is denoted by a blue dot). Thus, SNPs with a B allele frequency of one are apparent B/B homozygotes, SNPs with a B allele frequency of 0.5 are apparent A/B heterozygotes, and those with a B allele frequency of zero are apparent A/A homozygotes. Clearly, these plots show a contiguous region ~200 kb long with decreased copy number and apparent homozygosity (bounded by a red box). As we have demonstrated previously, this is indicative of a heterozygous genomic deletion [15]. Below these plots is a schematic of the two known genes affected by this deletion, *ITPR1* and *SUMF1*.

**Figure 3:**
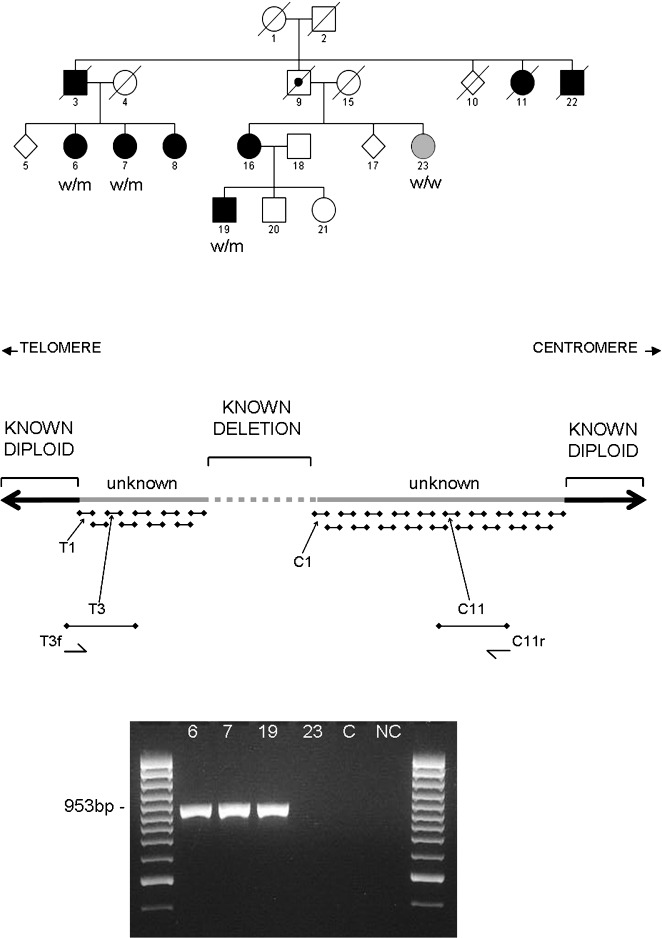
Mutation Analysis in the Australian SCA15 Family (Top) Pedigree of kindred. Filled symbols denote affected individuals; open symbols, unaffected individuals; grey symbol denotes unknown disease status; bulls-eye symbol denotes obligate carrier. w/w, wild-type at *ITPR1;* w/m, heterozygous carrier of the *ITPR1* deletion. (Middle) Schematic of primer pairs used to narrow the unknown regions between known deleted sequence and known diploid sequence at the SCA15 locus. Nine primer pairs (T1–T9) were used to amplify across the unknown region telomeric to the known deleted region; 19 primer pairs (C1–C19) were used to amplify across the unknown region centromeric to the known deleted region. All PCRs were carried out in the three affected family members. Analysis of these data narrowed the unknown region, and ultimately we were able to use primer T3f and C11r to amplify across the deletion breakpoint in the three affected family members, producing a fragment of 953 bp in affected individuals. (Bottom) Gel showing amplification product using primer pair T3f and C11r from affected pedigree members 6, 7, and 19; in pedigree member 23, with unknown disease affection status; in a neurologically normal control (C); and in a no template control (NC).

We obtained genomic DNA from three affected family members and one family member with unknown disease status from the kindred originally used to define and map SCA15 (family AUS1, of Australian Anglo-Celtic origin) [2]. We performed two experiments concurrently in three affected members of this family: sequence analysis of the coding exons of *ITPR1* and high-density genome-wide SNP genotyping. Sequence analysis failed to show any coding alterations segregating with disease or any alterations that were inconsistent with Mendelian patterns of inheritance within the family. However, visualization of log R ratio and B allele frequency metrics from the genome-wide SNP genotyping experiments clearly showed data consistent with a heterozygous genomic deletion across the first one-third of *ITPR1* and across the first half of a neighboring gene, *SUMF1* (Figure 2). This deletion was apparent in all three affected family members studied and absent from the family member with unknown affection status (Figure 3). The SNP data showed a deletion of between 188 kb and 210 kb in size; examination of SNPs at the flanking unknown regions of this deletion allowed us to delimit the borders of the deletion to 7.5 kb on the telomeric side of the deletion (between rs12634249 and rs793396) and ~14.4 kb on the centromeric side of the deletion (between rs4073665 and rs17709863). In an attempt to define whether this variation was a benign polymorphism we analyzed genome-wide SNP data at this locus, produced using the same genotyping chip, from 577 individuals of European descent who were either controls or individuals with an unrelated neurological disorder. We failed to find any deletions affecting the coding sequence of either gene, *ITPR1* or *SUMF1;* we did, however, identify a single individual with a possible heterozygous deletion approximately 6 kb in size within intron 40–41 of *ITPR1,* at least 5 kb away from exon 40. Given the location of this alteration it is unlikely to effect the expression or splicing of *ITPR1*.

In an attempt to fine-map the breakpoints of the disease-causing deletion we performed a series of experiments designed to refine the unknown intervals at the edges between definite deleted and definite diploid sequences. These data narrowed the unknown borders to ~4 kb on the telomeric side and ~7 kb on the centromeric side. We used all possible combinations of forward orientation primers designed within the newly defined telomeric boundary and of reverse orientation primers designed within the newly defined centromeric boundary in PCR assays in an attempt to amplify across the deletion in affected family members. Using PCR primers T3F and C11R, which should be more than 200 kb apart, we were able to amplify a fragment 953 bp in size using DNA from each of the three affected family members as template. Sequencing of this fragment revealed a deletion of 201,509 bp (Figure S3), removing the first three of the nine exons of *SUMF1* and the first ten of the 58 exons of *ITPR1*. We were unable to amplify the deletion-specific fragment in the family member of unknown affection status, or in 275 neurologically normal controls.

To further establish genetic deletion at *ITPR1* as the cause of SCA15 we analyzed two additional families with an inherited cerebellar ataxia similar to that described in the AUS1 family, ascertained through neurology clinics in London, United Kingdom. DNA extracted from probands from these two families (family H33 and family H27) were also analyzed using Illumina Infinium HumanHap550 genotyping chips. These experiments showed deletion at the SCA15 locus in all affected members assayed, from *SUMF1* through *ITPR1*. These mutations segregated with disease in these two families (Figure S3). A strategy similar to the one outlined above enabled us to sequence over the breakpoint in family H27 but not family H33. In the former, the deletion spans 344,408 bp, removing exons 1–3 of *SUMF1* and 1–44 of *ITPR1;* in the latter, we estimate that the deletion is 310 kb in length and that it removes exons 1–3 of *SUMF1* and exons 1–40 of *ITPR1*. The site of mutation is of interest, particularly the fact that in each of the three families the telomeric end of the deletion is anchored between exons 3 and 4 of *SUMF1;* sequence searches failed to identify any repeat sequences that might explain this phenomenon. With three cerebellar ataxia families segregating a *SUMF1–ITPR1* deletion, and this deletion not observed in a control population, we may reasonably conclude that the association is causal, and that the deletion is indeed the genetic basis of the disease, with SCA15 the diagnosis in the two British families as well as the original Australian family.

**Figure 4:**
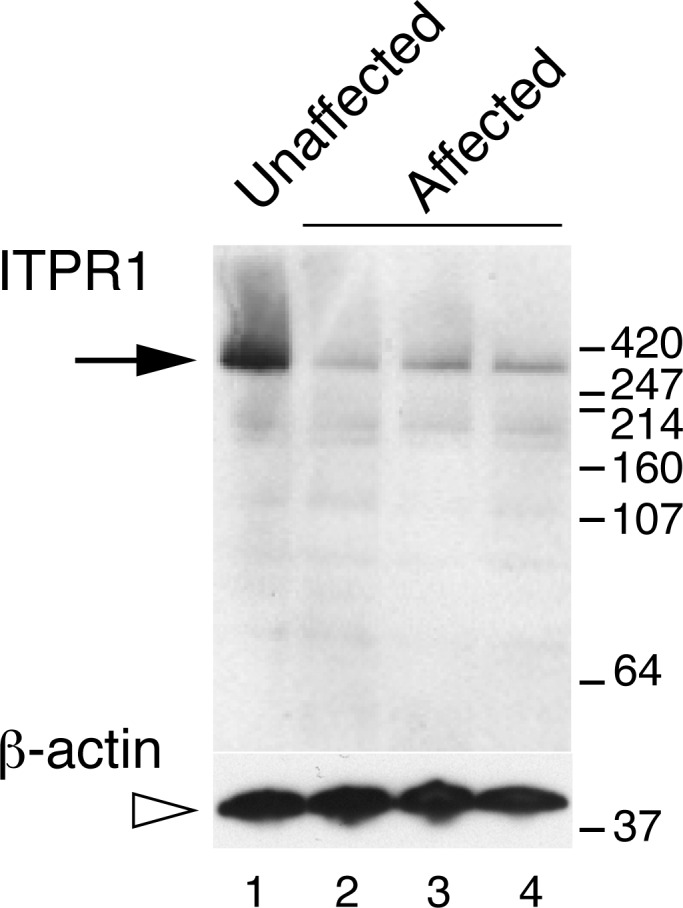
Western Blot Analysis of ITPR1 Protein Levels in EBV Immortalized Lymphoblasts from AUS1 Family Members Western blot performed to examine ITPR1 levels in EBV immortalized lymphocytes from AUS1 affected family members carrying the *ITPR1* deletion and from an AUS1 family member of unknown disease status who does not carry the deletion. Notably the samples from patients with *ITPR1* deletion show a dramatic decrease in ITPR1 levels. To demonstrate equal loading, these samples were diluted one in five, and the Western blot was repeated using an antibody against ACTB.

It is improbable that heterozygosity for the deletion of *SUMF1,* encoding sulfatase modifying factor 1, of itself causes or contributes to SCA15. Homozygous mutation of *SUMF1* results in autosomal recessive multiple sulfatase deficiency, a metabolic disorder characterized by hepatosplenomegaly, deafness, and developmental delay [6,7]. No co-occurrence of ataxia has been described in (heterozygous) parents of patients with multiple sulfatase deficiency. Conversely, mutation of *ITPR1* is biologically plausible as a cause of ataxia: the protein is highly expressed in Purkinje cells; as we have shown here, mice with mutation at this locus present with ataxia; and perturbed Ca^2+^ signaling has previously been implicated in the etiology of ataxia, notably in episodic ataxia type 2 and SCA6 [8]. In further support of this conclusion, analysis of protein levels of ITPR1 in Epstein-Barr virus (EBV) immortalized lymphocytes from affected and unaffected AUS1 family members revealed that all affected members showed a dramatic decrease in ITPR1 levels when compared with the family member without the deletion (Figure 4).

Itpr1 contains three domains, an N-terminal inositol triphosphate binding domain, a coupling domain, and a C-terminal transmembrane domain; it also contains two protein kinase A phosphorylation sites and an ATP-binding site. Itpr1 is coupled to Ca^2+^ channels and facilitates Ca^2+^ release from the endoplasmic reticulum after binding by the intracellular second messenger inositol 1,4,5-triphosphate [9]. Itpr1 is enriched in the Purkinje cells of the cerebellum [4]. ITPR1 mutations have more than one potential pathogenic mechanism. First, the disease may be a result of haploinsufficiency at *ITPR1;* this concept is consistent with the observation that heterozygous deletion leads to a later onset disorder in humans, whereas homozygous deletion in mice leads to an early onset disorder, able to be expressed within the much shorter life span of the mouse. Second, we cannot rule out the existence of an alternate start site for *ITPR1* that may result in a product that confers a pathogenic gain of function to the protein; however, Western blot analysis of cells derived from affected AUS1 family members, which was performed using an antibody raised against the C-terminal portion of ITPR1, failed to identify any disease-specific truncated protein products. Clearly, the identification of distinct *ITPR1* mutations underlying SCA15 will help elucidate the pathogenic mechanism of this disorder.

We show here the utility of investigating spontaneous mouse mutations in understanding human disease. Currently, the small number of aged *Itpr1^wt/Δ18^* animals precludes us from examining these mice for subtle signs and symptoms similar to those seen in SCA15 patients; however, these mice are clearly of interest to us as a potential model of SCA15. These data also demonstrate that genome-wide SNP assay can facilitate rapid detection of structural genomic mutations that may underlie disease. The data provided by these approaches provide compelling evidence that heterozygous deletion of *ITPR1* causes SCA15. Clearly, sequence analysis of *ITPR1* in potential SCA15 cases may provide additional insight into the disease, particularly if a stop mutation were to be identified; however, the mutational mechanism noted here means that standard sequencing approaches alone are insufficient to confidently rule out *ITPR1* mutation as a cause of disease: a comprehensive gene dosage approach is also required. Given that SCA16 and autosomal dominant congenital nonprogressive ataxia have both recently been mapped to regions overlapping with the SCA15 locus [10,11], *ITPR1* is a gene of importance for screening in these families. These data add weight to a role for aberrant intracellular Ca^2+^ signaling in Purkinje cells in the pathogenesis of spinocerebellar ataxia.

## Materials and Methods

### Genome-wide linkage in mice.

One hundred and twenty DNA fragments were amplified across the genome, each selected to contain one or more strain-specific SNPs that would differentiate between C57BL/6J and 129x1/SvJ inbred strains [12]. Each fragment was initially amplified in 11 affected mice and nine unaffected mice; genotype calling was performed by dye-terminator sequencing of these fragments. Linkage analysis using these data was performed using mlink [13], which revealed a positive linkage at Chromosome 6qE1, on the 129x1/SvJ background (two-point LOD score 5.13 at marker 20.MMHAP85FLG2). In an attempt to narrow the disease interval we performed backcross experiments that resulted in the generation of three additional affected mice. Genotyping of all affected mice across the disease-segregating interval revealed flanking recombinants and a candidate region of ~5 Mb, between markers D6Mit37 and 44.MMHAP85FLG5 (Figure S1). This region contains 16 genes and predicted transcripts.

### Identification of the underlying genetic lesion in mice.

Identification of similar phenotypes in mice linked to the 6qE1 interval was performed by literature searches. This revealed the *Itpr1^opt/opt^* mouse, in which disease is caused by homozygous deletion mutation of exons 43 and 44 of *Itpr1*. Primer pairs were designed to sequence each of the coding exons and at least 50 bp of each flanking intronic sequence of *Itpr1*. PCR amplification of each exon was performed using DNA from two affected mice as templates. The *Itpr1^Δ18/Δ18^* mutation was confirmed by sequencing in all affected mice (Figure S2).

Breeding experiments were performed between two female mice heterozygous for the current mutation (*Itpr1^wt/Δ18^*) and a male mouse heterozygous for the *Itpr1^opt^* mutation (*Itpr1^wt/opt^*). This resulted in two litters of mice with a total of four affected *Itpr1^opt/Δ18^* pups (from a total of 15; two of seven from first mating; two of eight from the second mating) with a phenotype indistinguishable from that of the *Itpr1^Δ18/Δ18^* and *Itpr1^opt^* ^/*opt*^ mice.

### Analysis of Itpr1 protein in mice.

We performed Western blot analyses using standard techniques with ECL detection kits (Amersham, http://www.amersham.com). Briefly, dissected whole brains from postnatal day 21 littermates were homogenized in a buffer containing 50 mM Tris-HCl, 150 mM NaCl, 1 mM EDTA, 1% Triton X-100, 1% sodium deoxycholate, 0.1% SDS, and a cocktail of protease inhibitors (Roche, http://www.roche.com). Homogenates were diluted appropriately, mixed with 4× reducing sample buffer, and loaded onto 4%–12% precast gradient gels (Novex, http://www.invitrogen.com) for SDS-PAGE and immunoblotting. The antibodies to Itpr1 (1:2,000) and Actb (1:5,000) were used as recommended by manufacturers.

### Immunohistochemistry.

Brains were isolated from 21-d-old mice, perfused with 4% paraformaldehyde in PBS, and post-fixed overnight in the same fixative. Brains were embedded in gelatin, and 35-μm sagittal sections were cut using a sliding microtome (NeuroScience Associates, http://www.neuroscienceassociates.com). Sections from wild-type, heterozygote, and homozygous brains were placed in the MultiBrain template. Sections were washed in 1× PBS prior to 1 h of incubation in block solution containing 1× PBS with 20% normal goat serum and 0.3% Triton X-100 (pH 7.4). Sections were incubated overnight at 4 °C in primary antibodies: affinity purified polyclonal Itpr1 antibody (1:2,000, Chemicon International, http://www.chemicon.com) and monoclonal anti-Calb1 antibody (1:6,000, Sigma-Aldrich, http://www.sigmaaldrich.com) diluted in carrier solution. Following extensive washes (in 6.0 ml of PBS, three times), sections were incubated with appropriate secondary antibodies (Alexa Fluor 555 goat anti-rabbit IgG and Alexa Fluor 488 goat anti-mouse IgG [Invitrogen, http://www.invitrogen.com]) for 1 h at room temperature. Sections were washed and mounted on glass slides in a buffered medium containing Mowiol (Calbiochem, http://www.emdbiosciences.com) as described earlier [14]. Sections were imaged using a laser scanning confocal microscope (LSM 510; Zeiss, http://www.zeiss.com). Imaging parameters (pinhole, detector gain, laser power) were optimized, and were kept constant for the wild-type, heterozygous, and homozygous mutant brains. Specificity of the Itpr1 antibodies was verified by preabsorption control experiments. Antibody dilutions were incubated for 24 h at 4 °C with the immunizing peptide. Tissue sections were incubated with the preabsorbed antibodies and processed as described above. Under these conditions, no staining above autofluorescence was detected.

### Analysis of ITPR1 in SCA15 patients.

DNA was extracted from EBV immortalized lymphocytes, derived from family members. The coding exons and at least 50 bp of flanking introns of *ITPR1* were PCR amplified and sequenced using dye-terminator sequencing (BigDye version 3.1; Applied Biosystems, http://www.appliedbiosystems.com). Sequence reactions were run on an ABI3730XP automated sequencer as per the manufacturer's instructions (Applied Biosystems). This analysis was performed in all three affected family members for whom genomic DNA was available (members 6, 7, and 19). Primer sequences and conditions are available upon request. Sequence data were analyzed using Sequencher (Gene Codes Corporation, http://www.genecodes.com). Genome-wide SNP genotyping was performed using Infinium HumanHap550 SNP genotyping chips as per the manufacturer's protocol (Illumina, http://www.illumina.com). This product assays 555,352 unique SNPs. Data were collected using the Illumina BeadStation scanner and data collection software. Genotypes were produced using the genotyping module of BeadStudio (version 2.3.25; Illumina), and log R ratio and B allele frequency were visualized using the genome viewer tool within this package. In order to rule out the possibility that the observed deletion within *ITPR1* was a benign copy number variant we examined log R ratio and B allele frequency metrics of HumanHap550 genotyping data at this locus from 577 individuals of Northern European descent from North America and Europe, produced by us as a part of an ongoing study.

In an attempt to narrow the unknown intervals flanking the deletion observed in family AUS1, we designed primers for 30 PCR amplifications that would generate overlapping fragments across the two bordering regions (primer sequence and conditions available upon request). There were ten primer pairs in the telomeric flanking region and 20 pairs in the centromeric flanking region (Figure S3). On average each product was ~750 bp in size, and amplifications were performed using genomic DNA from each of the three affected individuals (family members 6, 7, and 19). Dye-terminator sequencing of each product was performed using the forward and reverse primers designed for amplification; running and analysis of each fragment was performed as described above. Amplification of a fragment from a normal diploid genome was denoted by the presence of a heterozygous polymorphism; amplification of a fragment from a region of the genome harboring a heterozygous genomic deletion was inferred when homozygosity for the major allele and the minor allele were noted among the three affected family members (i.e., this is inconsistent with Mendelian inheritance in related individuals known to share a common haplotype).

Using the data from the experiments described above we were able to limit the size of unknown regions flanking the deletion to ~4 kb on the telomeric side and 7 kb on the centromeric side. All combinations of forward primers from the newly defined region flanking the deletion on the telomeric side with reverse primers from the newly defined region flanking the deletion on the centromeric side were used in PCR amplification reactions performed with DNA from the three affected family members and single unaffected family members. This experiment was performed in an attempt to amplify across the deleted fragment and define the exact breakpoint. A single fragment was obtained from the third forward primer from the telomeric side (T3f 5′-TGAATGCTCAATTTTCCAGC-3′) with the 11th reverse primer from the centromeric side (C11r 5′-GGGAAAATGGATAGAGGGTG-3′). The fragment, which is 953 bp in size, was sequenced as described above and compared to the current build of the human genome. A similar series of experiments was performed to identify the deletion breakpoints in families H27 and H33; we were able to amplify a 369-bp PCR product across the breakpoint found in affected members of family H27 using primer pair H27-11F 5′-GACCTCAAGAAGGCATGAATAC-3′ and H27-3R 5′-ATGGTGGCCAGGTACACAAG-3′ (Figure S4), but to date we have been unable to identify the breakpoint in family H33.

### Western blot analysis in SCA15 patients.

EBV immortalized lymphoblasts from three affected family members who carry the deletion and one family member without the mutation were used as a readily accessible source of protein; all samples came from members of family AUS1. Protein extraction was performed using lysis buffer containing 1× TBS, 1% Triton X-100, and a cocktail of protease inhibitors (Roche) with overnight lysis at −80 °C. Homogenates were diluted appropriately, mixed with 4× reducing sample buffer, and loaded onto 4%–12% precast gradient gels (NuPAGE, Invitrogen) for SDS-PAGE and immunoblotting. The antibodies to ITPR1 (1:1,000) and ACTB (1:5,000) were used as recommended by manufacturers.

## Supporting Information

Figure S1 Schematic of Genotyping Results across Mouse Chromosome 6 in Affected Mice Black squares are indicative of a C57BL/6J homozygous genotype; light grey squares, a 129x1/SvJ homozygous genotype; grey squares, a C57BL/6J 129x1/SvJ heterozygous genotype; white squares, undetermined genotype. The black box bounds a region of homozygous 129x1/SvJ genotypes that segregate with disease; thus, the critical region was determined to be between markers D6Mit37 and 44.MMHAP85FLG5.
(10 MB TIF)

Figure S2 Sequence of Exon 36 of *Itpr1* from Four Mice A wild-type homozygous C57BL/6J mouse (A), a wild-type homozygous 129x1/SvJ mouse (B), an affected 129x1B6 mice homozygous for the 18-bp deletion mutation (C), and an unaffected mouse heterozygous for the 18-bp deletion mutation (D). The deleted nucleotides are bounded by a green box.
(5.1 MB TIF)

Figure S3 Additional Families Harboring Deletion at the SCA15 Locus (A) Family H33; (B) family H27. Upper panel shows log R ratio and B allele frequency metrics generated from Infinium HumanHap550 arrays for an affected family member from each family. Log R ratio is the ratio of normalized, observed R to expected R for each SNP (each SNP is a blue dot) and thus serves as a surrogate of copy number at each locus. B allele frequency is a measure of the number of times the A or B alleles are detected at each locus (each SNP is denoted by a blue dot). Thus, SNPs with a B allele frequency of one are apparent B/B homozygotes, SNPs with a B allele frequency of 0.5 are apparent A/B heterozygotes, and those with a B allele frequency of zero are apparent A/A homozygotes. These plots show a contiguous region ~310 kb long (family H33) and ~350 kb long (family H27) with decreased copy number and apparent homozygosity indicative of a genomic deletion (shaded grey). The pedigrees below show the available family members assayed for these deletions, all of whom were affected and all of whom carried a deletion at this locus.
(6.8 MB TIF)

Figure S4 Deleted Regions Identified in Families AUS1 and H27 (Top) Family AUS1; sequence from the PCR product generated using primers T3f and C11r from genomic DNA from an affected family member. Red arrowhead denotes the deletion breakpoint; the deletion is 201,510 bp in length.
(Bottom) Family H27; sequence flanking deleted region. Green font indicates nucleotides telomeric to the deletion; blue font indicates nucleotides centromeric to the deletion. The deletion is 344,408 bp in length. Basepair positions are based on NCBI genome build 36 reference assembly.
(876 KB MB TIF).

### Accession Numbers

The OMIM (http://www.ncbi.nlm.nih.gov/entrez/query.fcgi?db=OMIM) accession number for *SUMF1* is 607939.

## Abbreviations

EBV: Epstein-Barr virus
SCA[number]: spinocerebellar ataxia [number]
SNP: single nucleotide polymorphism
